# Supplementary material for: HIVprotI: an integrated web based platform for prediction and design of HIV proteins inhibitors
Source: J Cheminform. 2018 Mar 9;10:12. doi: 10.1186/s13321-018-0266-y (PMC5845081; doi:10.1186/s13321-018-0266-y)
Supplement: Supplementary file 2 — Additional file 2. Source code of HIVProtI web server. [file 13321_2018_266_MOESM2_ESM.zip › HIVprotI_Source-code/hivprotI/weka-3-6-6/doc/weka/core/Trie.TrieIterator.html]

Trie.TrieIterator


---


|  |  |  |
| --- | --- | --- |
| **Overview** \| **Package** \| **Class** \| **Tree** \| **Deprecated** \| **Index** \| **Help** \| **Weka's home** | |  |
| **PREV CLASS**   **NEXT CLASS** | **FRAMES**    **NO FRAMES**     **All Classes** |  |
| SUMMARY: NESTED \| FIELD \| CONSTR \| METHOD | DETAIL: FIELD \| CONSTR \| METHOD |  |


---


## weka.core Class Trie.TrieIterator

```
java.lang.Object
  weka.core.Trie.TrieIterator
```

**All Implemented Interfaces:**: java.util.Iterator<java.lang.String>, RevisionHandler

**Enclosing class:**: Trie

---

``` public static class Trie.TrieIterator extends java.lang.Object implements java.util.Iterator<java.lang.String>, RevisionHandler ```

Represents an iterator over a trie

**Version:**
:   $Revision: 1.2 $

**Author:**
:   fracpete (fracpete at waikato dot ac dot nz)

---

**Constructor Summary**

`Trie.TrieIterator(Trie.TrieNode node)`
  
          initializes the iterator


| **Method Summary** | |
| --- | --- |
| `java.lang.String` | `getRevision()`             Returns the revision string. |
| `boolean` | `hasNext()`             Returns true if the iteration has more elements. |
| `java.lang.String` | `next()`             Returns the next element in the iteration. |
| `void` | `remove()`             ignored |

**Methods inherited from class java.lang.Object**

`equals, getClass, hashCode, notify, notifyAll, toString, wait, wait, wait`

**Constructor Detail**

### Trie.TrieIterator

```
public Trie.TrieIterator(Trie.TrieNode node)
```

:   initializes the iterator

    **Parameters:**: `node` - the node to use as root


**Method Detail**

### hasNext

```
public boolean hasNext()
```

:   Returns true if the iteration has more elements.

    :   **Specified by:**: `hasNext` in interface `java.util.Iterator<java.lang.String>`
    :   **Returns:**: true if there is at least one more element

---


### next

```
public java.lang.String next()
```

:   Returns the next element in the iteration.

    :   **Specified by:**: `next` in interface `java.util.Iterator<java.lang.String>`
    :   **Returns:**: the next element

---


### remove

```
public void remove()
```

:   ignored

    :   **Specified by:**: `remove` in interface `java.util.Iterator<java.lang.String>`

---


### getRevision

```
public java.lang.String getRevision()
```

:   Returns the revision string.

    :   **Specified by:**: `getRevision` in interface `RevisionHandler`
    :   **Returns:**: the revision


---


|  |  |  |
| --- | --- | --- |
| **Overview** \| **Package** \| **Class** \| **Tree** \| **Deprecated** \| **Index** \| **Help** \| **Weka's home** | |  |
| **PREV CLASS**   **NEXT CLASS** | **FRAMES**    **NO FRAMES**     **All Classes** |  |
| SUMMARY: NESTED \| FIELD \| CONSTR \| METHOD | DETAIL: FIELD \| CONSTR \| METHOD |  |


---
